# Evidence for cognitive vestibular integration impairment in idiopathic scoliosis patients

**DOI:** 10.1186/1471-2202-10-102

**Published:** 2009-08-25

**Authors:** Martin Simoneau, Vincent Lamothe, Émilie Hutin, Pierre Mercier, Normand Teasdale, Jean Blouin

**Affiliations:** 1 Faculté de Médecine, Université Laval, Québec, Canada; 2 Centre de recherche sur le vieillissement de Québec du Centre de recherche FRSQ - Hôpital Saint-Sacrement, Québec, Canada; 3 Neurobiology and Cognition Laboratory, CNRS and Aix Marseille University, Marseille, France

## Abstract

**Background:**

Adolescent idiopathic scoliosis is characterized by a three-dimensional deviation of the vertebral column and its etiopathogenesis is unknown. Various factors cause idiopathic scoliosis, and among these a prominent role has been attributed to the vestibular system. While the deficits in sensorimotor transformations have been documented in idiopathic scoliosis patients, little attention has been devoted to their capacity to integrate vestibular information for cognitive processing for space perception. Seated idiopathic scoliosis patients and control subjects experienced rotations of different directions and amplitudes in the dark and produced saccades that would reproduce their perceived spatial characteristics of the rotations (vestibular condition). We also controlled for possible alteration of the oculomotor and vestibular systems by measuring the subject's accuracy in producing saccades towards memorized peripheral targets in absence of body rotation and the gain of their vestibulo-ocular reflex.

**Results:**

Compared to healthy controls, the idiopathic scoliosis patients underestimated the amplitude of their rotations. Moreover, the results revealed that idiopathic scoliosis patients produced accurate saccades to memorized peripheral targets in absence of body rotation and that their vestibulo-ocular reflex gain did not differ from that of control participants.

**Conclusion:**

Overall, results of the present study demonstrate that idiopathic scoliosis patients have an alteration in cognitive integration of vestibular signals. It is possible that severe spine deformity developed partly due to impaired vestibular information travelling from the cerebellum to the vestibular cortical network or alteration in the cortical mechanisms processing the vestibular signals.

## Background

Idiopathic scoliosis is a complex deformation of the spine and it is the most common type of spine deformity. Its prevalence is about 2% to 3% in children aged between 10 to 16 years old and girls are at a higher risk than boys for severe progression. Although the etiopathogenesis of scoliosis is unknown, various factors have been identified that could be related to the etiology of scoliosis. Among these factors, it has been proposed that neurological mechanisms could be related to scoliosis [1]. Nonetheless, no clear-cut neurological tests either for diagnosing idiopathic scoliosis or for predicting its progression have so far been established.

Neurological deficits in idiopathic scoliosis have mostly been evidenced by studies examining the patient's motor behavior. For instance, several studies have demonstrated that compared to age-matched individuals, idiopathic scoliosis patients (ISP) show deficits in controlling their postural sway [e.g., [2]]. Because many idiopathic scoliosis patients show longer somatosensory cortical potentials (parietal N37) compared to healthy individuals, their balance instabilities could be associated with alteration in sensory signal processing [3-5]. Consistent with this hypothesis, impairment in balance control has been observed in ISP when sensory information is restored following transient deprivation of visual or somatosensory information (through vibration of the muscles around the ankle joint) [6]. The fact that postural sway increased when sensory information returned to normal leads to the suggestion that ISP have difficulty in dynamically adjusting the weight of the various sensory inputs to tailor the balance control commands.

Cases have been reported suggesting that the motor deficits in ISP may also be related to alterations in processing vestibular signals and in transforming these signals into motor commands. For instance, when the vestibular signals remain the only valuable source of information (i.e., in absence of vision and when ankle proprioception is altered through tendon vibration), body sway of ISP is aggravated compared to control participants [7-10]. These observations are in agreement with those published by Gauchard's et al. [11] revealing that ISP failed the balance control tasks that challenge the vestibular system.

While the deficits in sensorimotor transformations have been documented in ISP, little attention has been devoted to their capacity to integrate vestibular information for cognitive processing. According to electrophysiological and neuroimaging studies, the output of the vestibular apparatus projects either directly or indirectly to cortical regions of the brain essential for spatial processing (e.g., parietal cortex [12]). Furthermore, behavioral experiments conducted on healthy and neurological subjects have shown that perception of self movement and spatial orientation greatly rely on vestibular signal processing [13-16].

In addition to their documented deficits in transforming sensory signals into proper motor commands (e.g., [3,7,8,10]), here we tested whether ISP also show impairments in the processing of vestibular signals for cognitive spatial processes.

## Results

**Figure 1:**
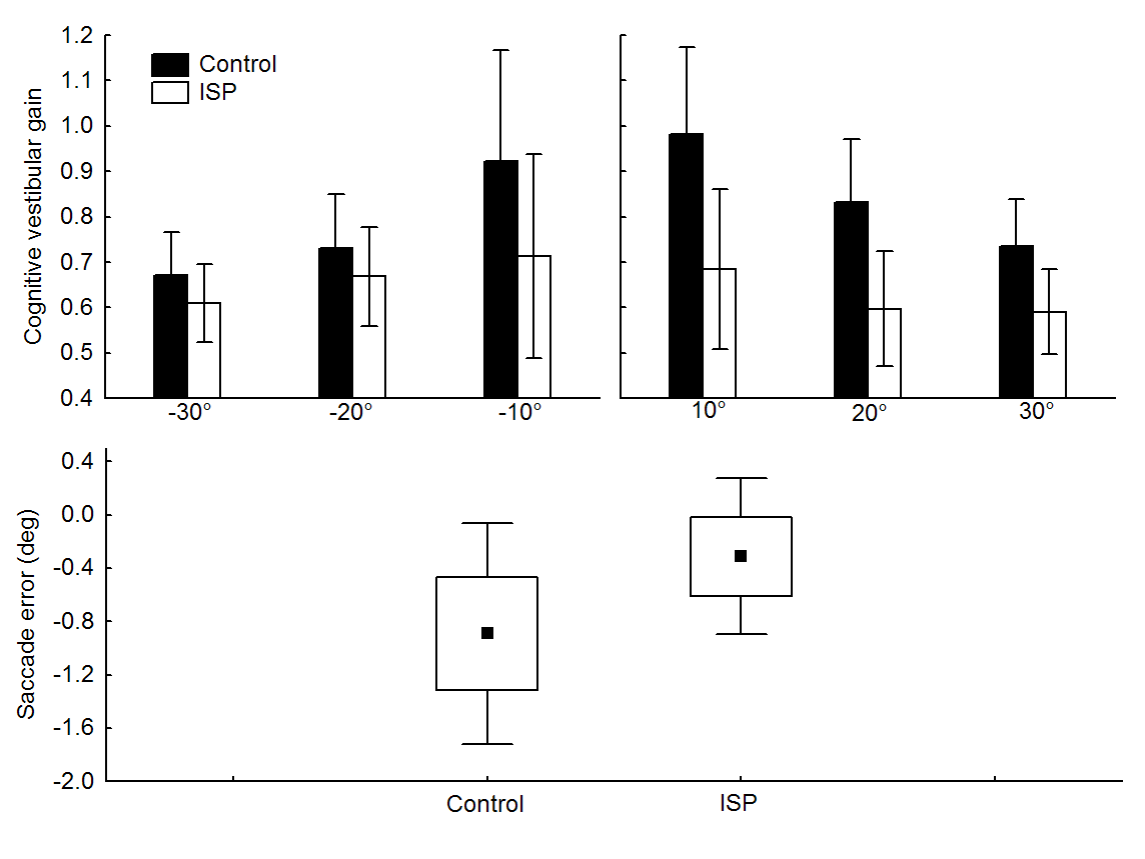
**experimental results**. Upper panel depicts both groups mean vestibular cognitive gain for chair rotations of ± 10°, ± 20° and ± 30°. The error bars represent ± 95% confidence interval. Lower panel presents both group means (black square = mean, boxes = standard error and, error bars = 1.96 × standard error) for the saccade accuracy in absence of chair rotation. Data for the ± 15° targets were pooled as no significant effect of direction was observed.

The analysis of the cognitive vestibular gain (Fig. 1 - upper panel) showed that both groups underestimated the whole-body rotation as demonstrated by their average which are below unity. Nonetheless, the ISP underestimated the magnitude of the whole-body rotation to a greater extent than the control participants (mean of 0.65 and 0.82 for ISP and Control, respectively; main effect of Group; F_1,20 _= 5.57, p < 0.05). The absence of significant interactions (i.e., Group by Direction or Group by Direction by Amplitude; ps > 0.05) suggests that, for both groups, the cognitive vestibular gains did not depend on the directions of the chair rotation nor on their amplitudes. On the other hand, the saccades produced by the ISP in the absence of whole-body rotation (Fig. 1 - lower panel) were accurate and their amplitude was not significantly different to that of control participants (t = 1.88, df = 20, p > 0.05).

The analyses revealed that the VOR gain (all participants results appear in table 1) of the ISP did not differ from that of control participants regardless of the rotation direction (no main effect of Group or Direction and no Group by Direction interaction: ps > 0.05). Importantly, the high percentage of the variance explained by the linear regression model for all participants confirms the efficiency of the model in quantifying the gain of the VOR for all participants and both rotations (no main effect of Group, Direction and no Group by Direction interaction: ps > 0.05).

**Table 1:** Gain of the vestibulo-ocular reflex and variance explained.

		**ROTATION +20°**		**ROTATION -20°**	
**ID**	**Group**	**VOR** **gain**	**Variance explained**	**VOR gain**	**Variance explained**

1	Control	0.92	0.90	0.91	0.88
2	Control	1.11	0.92	0.99	0.90
3	Control	0.92	0.92	0.83	0.79
4	Control	1.04	0.97	1.07	0.97
5	Control	1.00	0.96	0.93	0.92
6	Control	0.89	0.92	0.92	0.90
7	Control	1.03	0.99	1.07	0.97
8	Control	0.94	0.93	0.94	0.91
9	Control	1.19	0.96	1.13	0.95
10	Control	1.13	0.93	1.14	0.95
11	Control	1.08	0.96	1.09	0.94
12	Control	0.97	0.87	1.02	0.93

	**Mean**	**1.02**	**0.94**	**1.00**	**0.92**

1	ISP	1.03	0.95	0.97	0.93
2	ISP	1.06	0.93	1.08	0.91
3	ISP	1.00	0.97	1.10	0.97
4	ISP	1.03	0.92	0.89	0.89
5	ISP	0.87	0.85	1.07	0.90
6	ISP	0.98	0.95	1.10	0.94
7	ISP	0.93	0.91	1.01	0.95
8	ISP	1.08	0.93	1.06	0.96
9	ISP	0.98	0.98	0.98	0.97
10	ISP	0.94	0.89	0.99	0.97

	**Mean**	**0.99**	**0.93**	**1.02**	**0.94**

Note: for technical reason, we could not quantify the gain of the VOR of one control participant.

## Discussion

The present study confirms previous results revealing a good capacity of the control subjects to memorize and process vestibular signals to determine whole-body motions [13,15-21]. Compared to controls, however, the ISP underestimated the amplitude of the rotations suggesting impairment in their ability to memorize and process vestibular signals. None of the ISP showed asymmetry in perceiving leftward and rightward rotations. It is noteworthy that in absence of body rotation, ISP performed accurate saccades to memorized targets excluding any saccadic motor deficit. This observation substantiates previous results suggesting that the network assisting the control of vestibular memory-guided saccades is different from the one governing visual memory-guided saccades [22,23]. Moreover, the fact that ISP had normal vestibulo-ocular reflex gain confirms normal vestibular system in these patients.

Using the magnetic resonance imaging technique, Liu et al. [24] have recently demonstrated that the volume of several anatomical brain regions of ISP differs with respect to controls. For instance, compared to controls, ISP have larger parietal, temporal and frontal lobes. Despite the fact that no clear link can be established between brain volume and dysfunction, it is worth mentioning that these brain regions receive vestibular inputs and are involved in spatial attention and processing [12,13,22]. Therefore, the impairment of the ISP to memorize and process vestibular inputs could be due to an alteration of the cortical network processing vestibular signals. This would be consistent with the findings that, repetitive transcranial magnetic stimulation applied to the right posterior parietal cortex of healthy participants, impairs encoding of vestibular-derived displacement perception [25].

In addition, as idiopathic scoliosis is associated with melatonin signaling deficit [26], ISP impairment in perceiving whole-body rotation may witness alteration of the vestibulocerebellum functions. Indeed, although the human cerebellum is not considered as an endocrine system, the external zone of the cerebellar molecular layer has a high density of melatonin receptors [27]. Integrating vestibular, somatosensory and visual information, the vestibulocerebellum plays a key role in sensing head motion or head orientation in space and relative to gravity [28]. Remarkably, patients with midline cerebellar lesions underestimated the magnitude of their rotation despite normal vestibular function [29]. Therefore, it is likely that erroneous vestibular information going to the vestibular cortical network through cerebello-thalamocortical pathways prevents accurate computation of body rotation. However, not all processes mediated by the vestibulocerebellum would be altered as suggested by the ISP's accurate saccades to the memorized target in absence of body rotation. This may suggest that the rostral fastigial nuclei could be impaired by the melatonin signaling deficit in ISP. These nuclei, which are irresponsive to eye movements, are known as key processing centers for spatial orientation [30].

Manzoni and Miele [31] and Pompeiano et al. [32] have proposed that deficit in melatonin has an inhibitory effect on the vestibulospinal activity which could lead to abnormal activities of the cervical and axial muscles. In addition, impaired vestibular information from the cerebellum to the vestibular cortical network or alteration of this cortical network could also lead to alteration of back muscle activities. Therefore, vestibulospinal [31,32] as well as corticospinal abnormal activities may be part of the mechanisms leading to the onset and progression of scoliosis.

## Conclusion

The present results show that cognitive vestibular processing is impaired in ISP. It is possible that severe spine deformity developed partly due to impaired vestibular information travelling from the cerebellum to the vestibular cortical network or alteration in the cortical mechanisms processing of vestibular signals. The present study, however, does not allow one to make claims as to whether deficits in cognitive processing of vestibular signals should be considered as a potential factor leading to curve progression in scoliosis patients. Further studies may determine if this deficit and the melatonin signaling dysfunction in ISP can predict curve progression in patients with small spine deformity.

## Methods

### Subjects

Ten idiopathic scoliosis patients (9 female, mean age = 17.4, Cobb angle range = 28-51) and thirteen age-matched healthy individuals (11 female, mean age = 16.4) were tested and provided informed consent according to Laval University biomedical ethic committee. All the ISP had a right thoracic curve and a Riser sign greater than 3. Their curve had not progressed during the last year and no ISP wore bracing. No ISP had clinically detectable neurological disorders nor history of operative treatment or trauma to the brain. Furthermore, all participants had normal or corrected-to-normal vision.

### Method and Procedure

**Figure 2:**
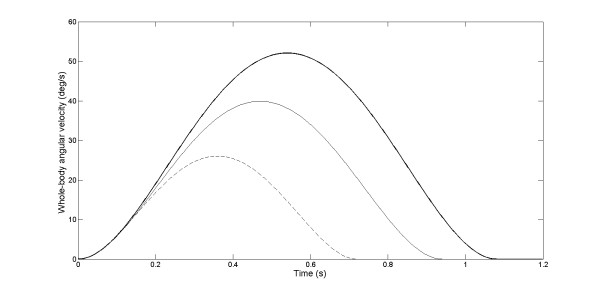
**whole-body angular velocity: cognitive vestibular condition**. Group mean time-series of the whole-body angular velocity for the chair rotation amplitude of 10° (dashed line), 20° (thin line) and 30° (thick line) are presented. Only counter-clockwise rotations are illustrated. Data are for the scoliosis group.

The participants sat in a completely dark room on a chair located in the center of a black cylinder with a radius of 1.5 m. The subjects were secured to the chair using a four-point belt and a chin support preventing head movement on the trunk during the rotations. The chair was manually rotated around the vertical axis using the handle attached to the rear of the chair. The rotations were measured with a potentiometer fixed at the centre of rotation of the chair. An array of LEDs placed behind the chair was used to display the magnitude of the chair rotation to be produced by the experimenter for each trial. The pseudo-randomly selected magnitudes of the whole-body rotation were 10°, 20° or 30°. The whole-body rotation was clockwise for half of the trials and counterclockwise for the other half. There were 8 trials for each rotation possibility. The chair was rotated following bell-shaped velocity profiles (Fig. 2) because it simulates the velocity profiles of natural head movements [33,34]. It is worth mentioning that similar whole-body dynamics were observed for both groups and direction (no main effects of Group or Direction and no Group by Direction by Amplitude interaction: ps > 0.05). As expected, however, the peak angular velocities were scaled according to the amplitude of the chair rotation (F_2,40 _= 12.21, p < 0.001).

Horizontal eye movements were recorded using electrooculography (Biomedica Mangoni model BM623). Chair rotations and eye movements were monitored at 200 Hz using a 12 bits A/D board (model LabMaster, Scientific Solutions, Mentor, OH, USA).

**Figure 3:**
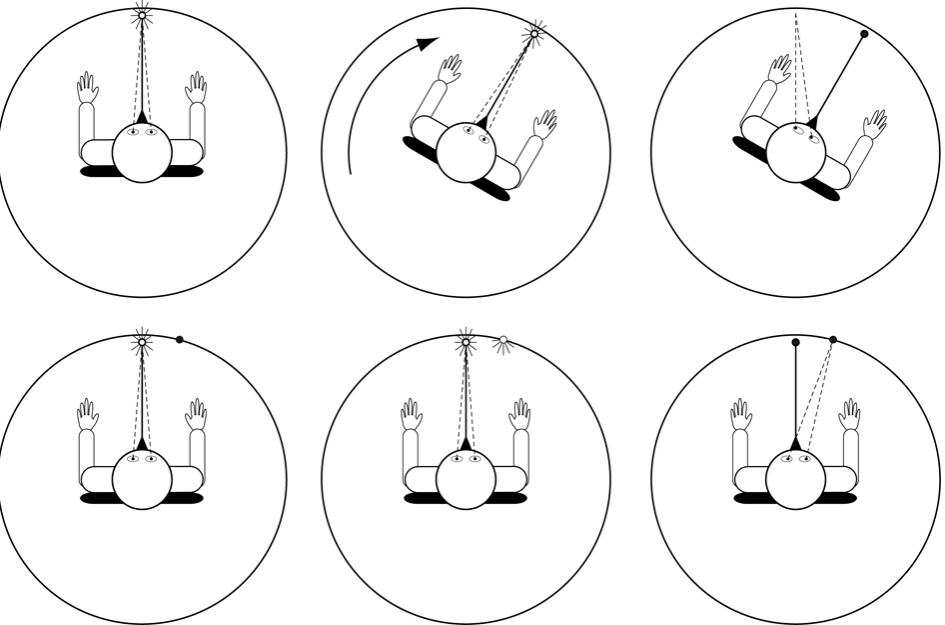
**experimental conditions**. Schematic of the experimental set-up: the upper panels depict the vestibular condition and lower panels the visual condition. Participants looked at a chair-fixed light (CFL) before being submitted to whole-body rotation (upper left panel). Then, the whole-body rotation stimulated mainly the horizontal semicircular canals (upper middle panel). Following the rotation, the CFL was turned off and the participants performed a saccade to the starting position (upper right panel). In the visual condition, the participants looked at the CFL (lower left panel). Then, the peripheral target lit up for 0.2 s (lower middle panel). Finally, following 1 s, the CFL was turned off and the participants performed a saccade to the peripheral target (lower right panel).

The experimental session consisted in a main experimental condition that tested the ability of the participants to process vestibular inputs (i.e., cognitive vestibular condition) and two control conditions; one that examined subjects' capacity in producing accurate saccadic eye movements (i.e., visual condition) and the other that controlled for possible alteration of the vestibular system (i.e., vestibulo-ocular reflex condition). In the cognitive vestibular condition, the participants were passively rotated. Eye movements were attenuated during body rotations by asking the subjects to look at a chair-fixed diode positioned straight-ahead. After the rotation, they produced a saccade in order to shift their gaze to their initial position ("vestibular memory-contingent saccade" paradigm [17]). The amplitude of the saccade (i.e., perceived body rotation amplitude) was measured at the offset of the first saccade (defined as the first instant that the eye velocity dropped below 5°/s after the saccade onset). Therefore, the participants had to process and memorize the vestibular signals generated during the whole-body rotation in order to produce a saccade of equal magnitude but in the opposite direction of the rotation (Fig. 3 - upper panels). In the visual condition, we verified whether the generation of saccadic eye movements differed between control participants and ISP in the absence of whole-body rotation. We did this by measuring the saccades produced towards a memorized peripheral target set at ± 15°. In this condition, the participants looked at a diode located straight ahead (fixation diode) while the peripheral target lit during 200 ms. Then, when the fixation diode was turned off (i.e., 1 s after the extinction of the peripheral target), the participants produced a saccade to the memorized visual target (Fig. 3 - lower panels). Participants performed 10 trials for each target direction. We calculated the final angular error, that is, the angular difference between the memorized peripheral target and the final angular position of the gaze. As for the cognitive vestibular condition, the amplitude of the saccade was measured at the offset of the first saccade.

Herman et al. [1] reported that some ISP have qualitative asymmetrical vestibulo-ocular reflex gain suggesting that the vestibular system may be impaired in ISP. For this reason we also quantified the gain of the vestibulo-ocular reflex of our patients and control participants. The amplitude of the chair rotation was ± 20° (10 trials per direction). The chair angular velocity was bell-shaped and the peak angular velocities were similar for both directions and groups (no main effects of Group or Direction and no Group by Direction interaction, ps > 0.05). The participants were instructed to keep their eyes on the fixation diode located straight ahead during the whole body rotation. For each trial, we calculated the gain of the vestibular-ocular reflex as the ratio between the slope of the linear regression of eye and chair velocities [35]. The fitting of the linear regression was performed from the onsets of the time-series of the eye angular velocity and the chair angular velocity until the peak eye angular velocity (i.e., group average: 208 ± 0.021 ms and 215 ± 0.028 ms for the ISP and control participants, respectively). To determine the accuracy of the fit of the linear regression, we calculated the coefficient of determination (r^2^).

### Statistical analysis

To quantify the cognitive vestibular gain, for each trial we divided the saccade amplitude (i.e., perceived angular rotation) by the actual whole-body rotation amplitude. Then, we performed a repeated-measures analysis of variance (Group by Amplitude by Direction). To ascertain that similar whole-body rotations were used for both groups in the cognitive vestibular condition, a repeated-measures analysis of variance (Group by Amplitude by Direction) was performed on the chair peak angular velocity. In the visual condition, we used a two-tailed t-test to verify if both groups produced different final saccadic angular error in absence of chair rotation. Finally, repeated-measures analyses of variance (Group by Direction) were used to assess the gain of the vestibulo-ocular reflex and the goodness of fit (i.e., variance explained by the model) of the linear regression model. All results were considered to be significant at the 5% critical level (p < 0.05).

## Competing interests

The authors declare that they have no competing interests.

## Authors' contributions

MS, NT and JB conceived the study. PM recruited the subjects and examined all the patients. VL and EH performed the data acquisition and analyses. EH and MS developed the Matlab programs to fit the linear regression model and to process data for quantifying the gain of the VOR. MS, JB and NT evaluated the data, supervised the data analyses and wrote the manuscript. All authors read and approved the final manuscript.
